# Editorial

**Published:** 1838-03-28

**Authors:** 


### MEDICAL EXAMINER.

PHILADELPHIA.

Wednesday, March 28, 1838.

In a former number* we took occasion, in referring to the recent work of M. QUETELET "*Sur l'Homme et le Development de des Facultés*," to speak of the effect of early marriages upon the human constitution. We stated our conviction that, the results of such precocious unions, were in general highly pernicious, that they tended ultimately to deteriorate the physical energies of the race, whilst immediately they entailed suffering and disease. In these views we were confirmed by M. QUETELET. In pursuing our remarks upon the subject of Marriage, we shall present our readers with some interesting statistical facts upon the influence of Marriage on Longevity, derived from a late number of the London *Lancet.* These are based upon observations made in France, Switzerland, and Holland, by DRS. CASPAR, OUDIER, and DEPARTIEUX. From *a priori* reasoning we might be induced to believe that the married state would in the majority of instances prove inimicable to the duration of life. We have elsewhere had occasion to notice the influences of moral cases upon longevity operating secondarily on the physical condition. The anxiety and cares of the husband and parent to provide for the wants of his partner and offspring, must act more or less prejudiciously. The bachelor untrammelled is enabled to pursue the selfish principle without check. He studies his own wants and follows the dictates of his own wishes; in short he exists for himself alone. The marriage state in the female inevitably entails consequences which we would naturally think, must lead to diminution of enjoyment, deterioration of health, and consequently to the shortening of life. These opinions are however contradicted by accurate investigations, as we shall presently show. The first table given proves "that the difference of life between married and unmarried females, on an average, is five years, or, to place the fact in a stronger

* Vide No. 4, p. 62–3.

point of view, a young woman of twenty years of age by marrying, adds nine years and a half to the probable duration of her life."

| Mean Duration of Life. | In Married Females. | In Unmarr'd Females. | Difference. |
|---|---|---|---|
| 20 years of age | 40.33 | 30.62 | years. 9.71 |
| 25 - - - - | 36.04 | 30.51 | 5.53 |
| 30 - - - - | 32.38 | 28.86 | 3.52 |
| 35 - - - - | 28.86 | 26.28 | 2.58 |
| 40 - - - - | 25.54 | 23.38 | 2.16 |

Departieux who composed a series of tables, comprising a total of 48,540 deaths, during a period of thirty years (from 1715 to 1744,) says, in a cursory manner, "it would appear that people live longer in a state of marriage than in one of celibacy. The number of married men who die after the age of 20 is nearly *one half* less than the number of bachelors who die at the same period; and for 48 married men, or widowers, who attain the age of 90, we find only six unmarried men reaching the same age. The number of single women who die after the age of 20, is about *four* times greater than that of married females, or widows, dying after the same period of life; and 14 virgins only (unmarried women,) arrive at the age of 90, for every 112 married women, or widows, who attain that advanced period of existence."

Of every hundred persons there die—,

| Period of Life. | Married Men. | Bachelors. | Married Women | Unmarried Women. |
|---|---|---|---|---|
| Years: | | | | |
| 20 to 30 | 2.8 | 31.3 | 7.7 | 28.0 |
| 30 to 45 | 18.9 | 27.4 | 20.3 | 19.3 |

Again of 100 persons there are alive,—

| Up to | Married Men. | Bachelors. | Married Women | Single Women. |
|---|---|---|---|---|
| 30 years | 97.2 | 68.7 | 92.3 | 72. |
| 45 - - | 78.3 | 41.3 | 72.0 | 52.7 |
| 60 - - | 48.1 | 22.6 | 49.4 | 37.2 |
| 70 - - | 27.2 | 11.1 | 29.2 | 23.7 |

The above tables present a very remarkable difference in the comparative mortality of married and unmarried men, between the ages of 20 and 30. We shall not, however, insist much upon this difference for reasons which must be familiar to all who are in the habit of estimating the true value of calculations of this kind; men, taking the masses, seldom marry before they have acquired a certain position in the world, and attained a degree of comfort or even ease, which, as is well known, contribute very materially to the diminution of mortality; but looking to the period comprised between thirty and forty-five years a period during which men generally marry, we still find a difference of mortality which is considerably in favour of those who have taken unto themselves wives; after the forty-fifth year, this proportion on the side of married men goes on increasing; for the tables show us that, taking 100 married and 100 unmarried individuals, the number of those who live beyond the age of 45 is greater by 36.8 in the former class than in the latter.

This is a fresh proof of the favourable influence which marriage exercises over the duration of human life at the earlier periods of existence; at a later period its influence becomes still more manifest, since for 11 bachelors who live beyond the age of 70 we find no less than 27 married men.

According to Biches' calculation, of 100 individuals there die,—

| From | Married Men. | Single Men. | Married Women | Single Women. |
|---|---|---|---|---|
| 20 to 30 | 3.6 | 33.1 | 4.7 | 26.5 |
| 30 to 45 | 17.9 | 27.1 | 16.5 | 24.5 |
| 45 to 60 | 29.2 | 15.6 | 22.6 | 19.2 |

A Mr. Rickman has attempted in the *London Medical Gazette* to refute what has been called by an esteemed contemporary, this "libel upon bachelors and maids," but we think the document just quoted proved conclusively that the balance is against the advocate for "single blessedness," whilst it sustains our views of precocious marriages, and the prudence of restraining the consummation of connubial engagements until the physical man has attained its maximum of energy.

